# In-Situ Monitoring the SERS Spectra of para-Aminothiophenol Adsorbed on Plasmon-Tunable Au@Ag Core–Shell Nanostars

**DOI:** 10.3390/nano12071156

**Published:** 2022-03-31

**Authors:** Yan Ke, Bin Chen, Mengen Hu, Ningning Zhou, Zhulin Huang, Guowen Meng

**Affiliations:** 1 Key Laboratory of Materials Physics and Anhui Key Laboratory of Nanomaterials and Nanotechnology, Institute of Solid State Physics, HIPS, Chinese Academy of Sciences, Hefei 230031, China; keyancosb@163.com (Y.K.); bchen@issp.ac.cn (B.C.); mengenhu@issp.ac.cn (M.H.); 2 University of Science and Technology of China, Hefei 230026, China; 3 Department of Chemical and Materials Engineering, Hefei University, Hefei 230601, China; zhnn@hfuu.edu.cn

**Keywords:** plasmon-driven photocatalysis, SERS, nanostars, resonant excitation

## Abstract

Plasmon-induced photocatalysis on noble metal surfaces has attracted broad attention due to its application in sunlight energy conversion, while the selectivity of plasmonic platforms remains unclear. Herein, we present the controlled plasmon-mediated oxidation of para-aminothiophenol (*p*-ATP) by employing Au@Ag core–shell nanostars with tunable tip plasmons in visible–near-infrared range as reactors. In-situ Raman measurements indicate that Au@Ag core–shell nanostars essentially promote the conversion of *p*-ATP to 4,4′-dimercaptoazobenzene (DMAB) due to hot carriers excited by localized surface plasmon resonance. Au@Ag nanostars with plasmon modes under resonant light excitation suggested higher catalytic efficiency, as evidenced by the larger intensity ratios between 1440 cm^−1^ (N=N stretching of DMAB) and 1080 cm^−1^ shifts (C–S stretching of *p*-ATP). Importantly, the time-dependent surface-enhanced Raman scattering spectra showed that the conversion efficiency of *p*-ATP was mainly dictated by the resonance condition between the tip plasmon mode of Au@Ag core–shell nanostars and the excitation light, as well as the choice of excitation wavelength. These results show that plasmon bands of metal nanostructures play an important role in the efficiency of plasmon-driven photocatalysis.

## 1. Introduction

The interaction between light and metals leads to the localized surface plasmon resonance (LSPR) effect on noble metallic nanostructures, including light scattering/absorbance [1,2], excitation of transient hot electrons and holes [3,4], plasmon-induced resonance energy transfer [5,6], near-field enhancement [7], as well as thermal effect [8]. The unique optical properties of metal nanostructures render them promising candidates in the areas of solar cells [9,10], imaging [11,12], photothermal therapy [13,14], seawater desalination [15], plasmon-driven photocatalysis [16,17], and surface-enhanced Raman scattering (SERS)-based signal amplification of trace molecules [18,19]. Plasmon-driven photocatalysis has attracted lots of attention due to its potential application in sunlight energy conversion [16]. In this process, when metal nanostructures absorb photons, they behave as highly effective light-trapping centers generating collective oscillation of free electrons. In a time scale of 10~100 fs, interband electron transitions are excited in the plasmon decay, leading to the nonequilibrium state electrons and holes [3]. The hot carriers then tunnel out of the metal surface, resulting in reduction of adsorbed molecules on the metal surface by accepting such photo-excited electrons or in molecule oxidation by transferring electrons into adjacent metal nanostructures. In SERS-based detection, enhancement of the localized electromagnetic field of metallic nanostructures by the LSPR effect will enhance the Raman scattering signals of adsorbed molecules by 10^4^~10^14^ times [20,21], thus, providing vibrational fingerprints of trace molecules. In this regard, specific plasmonic nanostructures possessing both catalytic activity and SERS enhancement will drive photocatalytic reactions; the catalytic reaction processes can be monitored by time-dependent SERS spectroscopy, while the catalytic activities of the utilized material can also be evaluated.

Therefore, a lot of research is emphasized on the design of noble metal and metal/semiconductor nanostructures to qualitatively testify the plasmon-driven photocatalysis [22]. The frequently used metal plasmonic catalytic materials [16] include the following: Au, Ag, Cu, Al, Pt, Pd, Ru [23], and Rh [24], among which Au and Ag exhibit high SERS enhancement. For this reason, Au and Ag nanostructures, as well as their composite nanostructures with semiconductors, such as Ag microflowers [25], nanoporous Au nanoarrays [26], Au–Pd nanoparticles [27], Ag–ZnO–CeO_2_ heterostructures [28], Au/Pt/Au core–shell nanoraspberries [29], and TiO_2_–Au nanoparticles [30], were employed as reactors to investigate both plasmon-mediated catalytic reactions and evolutions of SERS spectra. A typical example is the plasmon-driven oxidation of para-aminothiophenol (*p*-ATP) into 4,4′-dimercaptoazobenzene (DMAB) [31]. In this process, the SERS spectra of *p*-ATP adsorbed on Au and Ag nanoparticles present a set of abnormal peaks at 1148 cm^−1^, 1390 cm^−1^, and 1432 cm^−1^ shifts, being ascribed to the evolution of two amine groups (-NH_2_) into an azoxy group (-N=N-), forming a new molecule—DMAB [32]. Further study shows that at the Au/air or Ag/air interface, the plasmon-excited hot electrons can transfer to the adsorbed O_2_ molecules and generate O_2_^−^ radicals, which then participate in the oxidation of *p*-ATP into DMAB [30].

Recently, Au nanostars caught much attention in the plasmon community [33,34,35] due to the following reasons: (1) the sharp spikes on Au nanostars can generate a strong LSPR effect, leading to large SERS enhancement and efficient hot electrons/holes which can transport to adsorbates on the tips, promoting catalytic reactions; (2) the plasmon of Au nanostars can be fitted as the core mode (typically in the range of 520~560 nm) and the tip mode (typically in the range of 600~900 nm), with the latter being tunable in Vis–NIR range by adjusting the sharpness and length of the nanotips [36,37]; (3) Au is stable and biocompatible. For example, Sousa-Castillo et al. revealed that Au nanostars endowed TiO_2_ with a strongly enhanced photocatalytic efficiency compared to Au nanospheres or nanorods [35]. However, Ag tends to affiliate more oxygen molecules than Au to generate more O_2_^−^ radicals through electron transfer, which are beneficial for subsequent catalytic reactions such as oxidation of *p*-ATP. More importantly, Ag has stronger plasmon effect and smaller work function than Au (4.26 eV vs. 5.20 eV) [38], making Ag more promising in plasmon-driven catalysis. However, the strong diffusion of Ag atoms makes it difficult to form a stellate shape. To achieve superhigh plasmonic properties enhanced by both spike structure and Ag material, Ye et al. synthesized spiky hollow Ag–Au nanostars composed of 90% Au, 10% Ag, and a few nanometers thick Ag-rich surface layer in one step for enhanced catalysis and single-particle SERS [39]. Besides, Kaur and coworkers reported anisotropic-shaped bimetallic Au/Ag nanostars prepared by coating a thin Ag layer on the surface of Au nanostars for plasmon-driven photocatalytic oxidation of *p*-ATP into DMAB [40]. The core–shell Au@Ag nanostars showed much higher photocatalytic efficiency compared to Au nanostars alone, which could rapidly trigger the dimerization of *p*-ATP even when the concentrations of Au@Ag nanostars were as low as 72, 36, and 9 pM under acidic, neutral, and basic conditions, respectively. Nevertheless, the selective control mechanism of catalytic activity, as well as the reaction kinetics by means of plasmon effect from noble metals were few studied in above works and remain unclear. In order to monitor the plasmon-mediated catalysis, the plasmon of Au and Ag nanostructures across the visible–near-infrared (Vis–NIR) region needs to be regulated priorly to cater for different excitation wavelengths. Fortunately, liquid-phase chemistry has been used to tune the shape and size of the nanostructures so that precise control over the plasmon of Au and Au@Ag nanostars can be realized [41].

Based on the above understanding, herein, we demonstrate Au@Ag core–shell nanostars with tunable plasmon modes in the Vis–NIR region and a selective plasmon-driven photochemical reaction during the conversion of *p*-ATP into DMAB. We show that Au nanostars in the size range of 30~90 nm can be fabricated by seed-mediated liquid-phase chemistry, demonstrating two plasmon modes around 520 nm (core mode) and Vis–NIR range (tip mode, up to 890 nm). After in-situ coating with a ~2 nm Ag shell, the plasmon modes blue shifted 30~50 nm accordingly, but the stellate morphology still retained. Under illumination of different excitation wavelengths (532 nm, 633 nm, and 785 nm) the bare Au nanostars demonstrated overall stable SERS spectra of *p*-ATP, indicating poor plasmon-driven catalytic activities. Nevertheless, the composite Au@Ag core–shell nanostars were able to induce SERS spectral evolution of *p*-ATP. More importantly, when samples were excited within the resonance range of the tip plasmon modes, SERS intensities located at 1148 cm^−1^, 1390 cm^−1^, 1440 cm^−1^, and 1578 cm^−1^ of DMAB were essentially significant, demonstrating enhanced plasmon-driven catalytic effect. The composite Au@Ag core–shell nanostars showed potential in the conversion of energy and plasmon-driven catalytic reactions.

## 2. Results and Discussion

### 2.1. Characterization of Bare Au Nanostars

**Figure 1:**
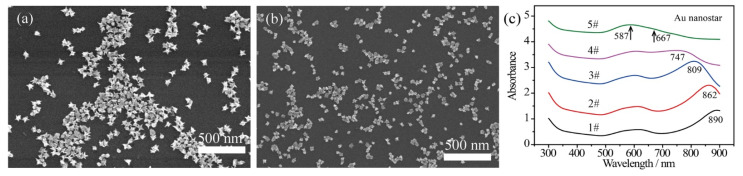
SEM images of bare Au nanostars prepared with addition of (**a**) 45 μL and (**b**) 450 μL Au seeds in the precursor, showing immediate size change. (**c**) UV–Vis–NIR absorbance spectra achieved with varied Au seeds of 45 μL, 129 μL, 240 μL, 450 μL, and 750 μL for sample 1# to sample 5#, respectively.

The bare Au nanostars were fabricated with a modified liquid-phase chemistry approach, as described previously [42]. The size of the bare Au nanostars was controlled by tuning the concentration of injected Au seeds and maintaining the concentration of chloroauric acid in the precursor. Herein, the amount of chloroauric acid was 0.25 mL (50 mM) and the dosages of added Au nanoseeds were 45 μL, 129 μL, 240 μL, 450 μL, and 750 μL, respectively. Typical scanning electron microscopy (SEM) images in Figure 1a,b show that the size of Au nanostars (defined as the moderate horizontal length) was estimated to be 90 nm (Figure 1a, 45 μL Au seeds) and 40 nm (Figure 1b, 450 μL Au seeds). Each Au nanostar was featured by 6~9 sharp spikes with length of 10~40 nm depending on the size of the Au nanostars. With the increase of Au seed concentration, the particle size decreased. In this dynamic growing process, the reduced Au atoms accumulated on the surface of the spherical Au seeds, while the chains of surfactant PVP k15 selectively bound to the {111} crystal facets [43], leading to the anisotropic growth of Au spikes. With varying addition of Au seeds, the length of the Au spikes was adjusted from ~35 nm (45 μL Au seeds) to 10~15 nm (450 μL Au seeds).

The plasmon bands of the bare Au nanostars with varied sizes reveal a tunable range from 587–890 nm (Figure 1c). It can be seen that the core plasmon mode of Au nanostars blue shifts slightly to around 590 nm due to the size variation of Au cores. The tip plasmon mode shifts from 890 nm to 667 nm due to the decreased length of the Au spikes. Besides, with the spheroidization of the Au nanostars (in accordance with small Au nanostars achieved at high concentration of Au seeds), the LSPR band of the Au nanotips and the core plasmon mode degenerated as a broad peak, as presented by curve 5# in Figure 1c.

### 2.2. Characterization of Composite Au@Ag Core–Shell Nanostars

**Figure 2:**
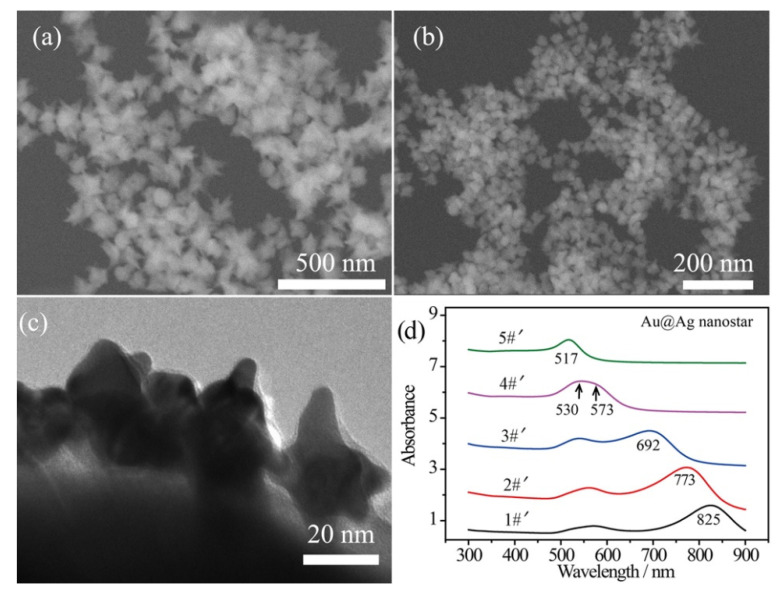
SEM images of the Au@Ag core–shell nanostars obtained by coating ~2 nm Ag shell on (**a**) 90 nm and (**b**) 40 nm Au nanostars. (**c**) TEM image showing a thin Ag shell on the surface of 40 nm Au nanostars. (**d**) UV–Vis–NIR absorbance spectra of Au@Ag core–shell nanostars prepared by coating Ag shell on samples 1#~5#, demonstrated in Figure 1c, showing blue-shifted tip plasmon mode.

In order to enhance the plasmonic effect, Ag shells were wrapped on the surface of the bare Au nanostars by a polyol reduction process, while the thickness of the Ag shells was tailored by the amount of Ag element [44]. An ultrathin Ag shell (~2 nm) was wrapped on the Au surface with a small amount of AgNO_3_ solution (125 μL, 16 mg/mL) added in one shot of Au nanostar product. In this case, the spiky morphology can be maintained without over spheroidization with such 2 nm Ag shell. Figure 2a,b shows the SEM images of the composite Au@Ag core–shell nanostars, corresponding to the 90 nm and 40 nm inner Au nanostars, respectively. Additionally, a transmission electron microscopy (TEM) image of Ag-coated 40 nm Au nanostars reveals the thickness of the Ag shell to be ~2 nm and the radius of curvature to be 5~8 nm (Figure 2c). The broad LSPR band in curve 4#′ can be decomposed as the core plasmon mode around 530 nm and the tip mode at 573 nm for the Ag-coated 40 nm Au nanostars (Figure 2c). Generally, the LSPR bands of Au@Ag core–shell nanostars blue shifted due to the decreased sharpness of the tips and the presence of Ag. This was especially remarkable for curve 5#′, in which the LSPR band shifted to 517 nm.

### 2.3. Plasmon-Mediated Oxidation of p-ATP by Bare Au Nanostars

**Figure 3:**
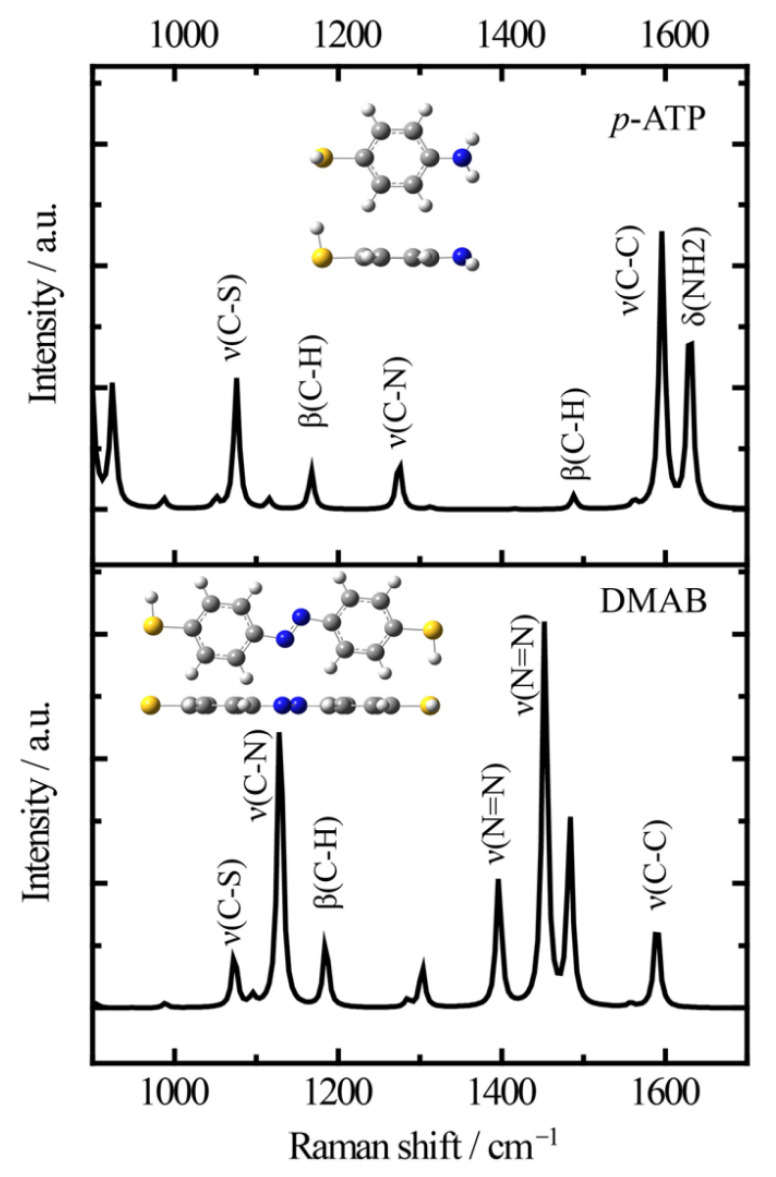
Simulated Raman spectra of *p*-ATP and DMAB molecules and the optimized molecule structures (B3LYP/6–31+G(d)).

Firstly, it is necessary to assign the vibrational modes of *p*-ATP and DMAB molecules, so the DFT theory included in the Gaussian 09W software was used to simulate the optimized structures and Raman spectra of *p*-ATP and DMAB molecules. The hybrid density functional B3LYP method with 6–31G/(d) basis set was used. A scale factor of 0.9613 was used to correct the basis set superposition errors. Figure 3 shows the optimized molecule structures of *p*-ATP and DMAB molecules. The main fingerprints of *p*-ATP are located at 1076 cm^−1^ (C–S stretching), 1167 cm^−1^ and 1488 cm^−1^ (C–H in-plane bending), 1274 cm^−1^ (C–N stretching), 1596 cm^−1^ (parallel C–C stretching), and 1630 cm^−1^ (amine scissoring). As for DMAB molecules, the C–S stretching mode slightly shifts to 1073 cm^−1^, and the C–H in-plane bending modes appear at 1129 cm^−1^ and 1185 cm^−1^. The bands at 1396 cm^−1^ and 1453 cm^−1^ can be assigned to the N=N stretching. To be mentioned, considering the basis set superposition errors involved in the simulation process, these vibrational assignments are generally in agreement with subsequent actual SERS measurements.

Herein, to examine the plasmon-driven catalytic activity of Au nanostars, Au nanostar substrates were immersed in *p*-ATP solutions for 4 h to obtain a self-assembled molecule layer on the Au nanostar surface. Xe light was used to simulate sunlight and illuminate the Au nanostar substrates. The output excitation wavelengths were set at 515 nm, 633 nm, and 785 nm to cater for different plasmon bands of Au nanostars. The power density projected on the Au nanostar substrates was tuned and limited to ~2 mW/cm^2^ to avoid possible thermal-induced chemical reactions on the Au nanostars.

**Figure 4:**
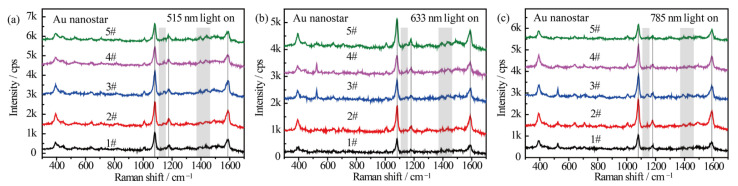
SERS spectra of *p*-ATP adsorbed on Au nanostars with different tip plasmon modes after illumination with (**a**) 515 nm, (**b**) 633 nm, and (**c**) 785 nm monochromatic lights for 1 h. SERS spectra were measured at 785 nm excitation line and accumulation time was 1 s.

Figure 4 shows the wavelength-dependent SERS spectral evolution of *p*-ATP adsorbed on Au nanostars. The main peaks in the SERS spectra at 1080 cm^−1^ (C–S stretching), 1179 cm^−1^ (C–H in-plane bending), and 1593 cm^−1^ (ring C–C stretching) can be assigned to the vibrational fingerprints of *p*-ATP (highlighted by black lines). Nevertheless, the fingerprint peaks of DMAB at 1148 cm^−1^, 1390 cm^−1^, and 1440 cm^−1^ were extremely weak (highlighted by grey rectangles). As the azo-coupling reaction is characterized by the existence of nitrogen double bonds, hence the catalytic activity was evaluated by comparing the intensity ratio (RI) of the relative intensities of the main bands at 1440 cm^−1^ (N=N stretching of DMAB, in correspondence to the 1453 cm^−1^ shift in Figure 3) and 1080 cm^−1^ (C–S stretching of *p*-ATP) [30]. With the 515 nm, 633 nm, and 785 nm radiation of Xe light for 1 h, very weak peaks of DMAB appeared from all 5 samples (Figure 4a–c). The counterpart *R_I_* values were all calculated to be less than 0.05, indicating that few *p*-ATP molecules transformed to DMAB. In addition, all the samples demonstrated similar SERS intensities and peak locations of *p*-ATP although they have different plasmon bands. This is quite different from the case of Ag-related samples, which will be discussed later. The low plasmonic catalytic activity from Au nanostructures may be ascribed to the limited amount of plasmon-induced O_2_^−^ radicals on the Au nanostars because O_2_ molecules are difficult to affiliate on Au surfaces, as well as the high work function of Au that makes it difficult for electrons to escape from the material`s surface [45]; therefore, few *p*-ATP molecules were oxidized into DMAB molecules.

### 2.4. Plasmon-Driven Oxidation of p-ATP by Au@Ag Core–Shell Nanostars

**Figure 5:**
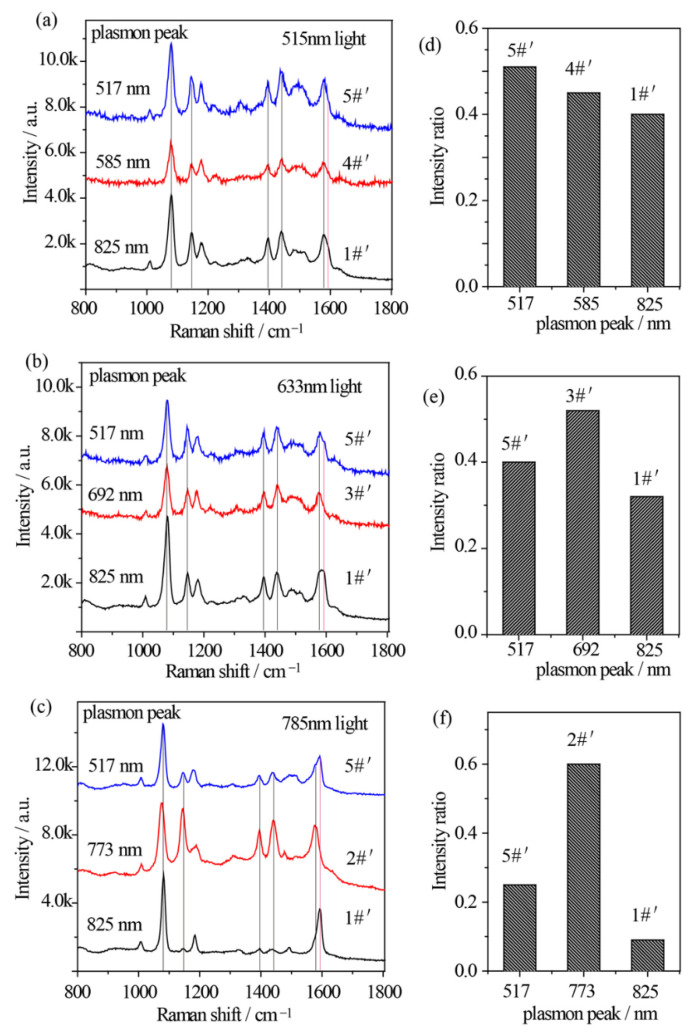
(**a**–**c**) SERS spectral evolutions of *p*-ATP adsorbed on Au@Ag core–shell nanostars with varied tip plasmon modes after excitation with (**a**) 515 nm, (**b**) 633 nm, and (**c**) 785 nm monochromatic lights for 1 h, indicating the formation of DMAB molecules. (**d**–**f**) Relative intensity ratios between the 1440 cm^−1^ shift (N=N stretching of DMAB molecule) and the 1080 cm^−1^ shift (C–S stretching of *p*-ATP molecule) correspond to (**a**–**c**).

The relationship between the plasmon band, excitation wavelength, and catalytic kinetics of Au nanostars after Ag shell coating is quite different. Similarly, 515 nm, 633 nm, and 785 nm excitations from Xe light were used to excite the *p*-ATP molecules adsorbed Au@Ag core–shell nanostars with different plasmon bands. Specially, sample 5#′, 3#′, and 2#′ were selected to drive the oxidation of *p*-ATP under illumination of 515 nm, 633 nm, and 785 nm monochromatic lights, respectively, which were close to the LSPR bands of the composite Au@Ag core–shell nanostars. Meanwhile, sample 1#′ (plasmon peak at 825 nm) and sample 4#′ (plasmon peak at 573 nm) were selected for comparison under illumination of 515 nm monochromatic light, and sample 1#′ and sample 5#′ (plasmon peak at 517 nm) were selected for comparison under illumination of 633 nm and 785 nm monochromatic lights. As shown in Figure 5a–c, the fingerprint peaks of *p*-ATP at 1080 cm^−1^, 1179 cm^−1^, and 1593 cm^−1^ were observed, meanwhile new peaks appeared at 1148 cm^−1^ (C–H in-plane bending), 1390 cm^−1^ (N=N stretching), 1440 cm^−1^ (N=N stretching), and 1578 cm^−1^, which can be assigned to the vibrational fingerprints of DMAB, being in agreement with the DFT simulation results. It clearly shows that all 3 samples of Au@Ag core–shell nanostars with different plasmon bands generally realized partial conversion of *p*-ATP to DMAB under 515 nm, 633 nm, and 785 illuminations with higher conversion rates in comparison with that of Au nanostars.

In addition to the lower work function of Ag and higher affinity of Ag to O_2_, the electronic charges may transfer from core Au atoms to shell Ag atoms and lead to an increase in the electron density on the surface of Au@Ag core–shell nanostars, largely improving catalytic activity [46]. With 515 nm illumination, due to the superposition between the 515 nm wavelength and the plasmon band of sample 5#′, *R_I_* value was estimated to be 0.51, larger than that of sample 1#′ (*R_I_* = 0.40) and 4#′ (*R_I_* = 0.45) (Figure 5a,d). Besides, with 633 nm radiation, *R_I_* value of sample 3#′ was estimated to be 0.52, larger than that of sample 1#′ (*R_I_* = 0.32) and 5#′ (*R_I_* = 0.40) (Figure 5b,e), owing to the minimum gap between the plasmon band of sample 3#′ and the 633 nm wavelength. In addition, Figure 5c clearly demonstrates that with 785 nm excitation on sample 2#′, the Raman bands of DMAB at 1148 cm^−1^ and 1440 cm^−1^ obviously become pronounced with an increased *R_I_* value of 0.6 (Figure 5f), indicating an increased conversion rate of *p*-ATP. Interestingly, unlike the slight variation in Figure 5d,e, both sample 1#′ and 5#′ demonstrated a relatively low *R_I_* value, possibly owing to the lowest photon energy of 785 nm light in comparison with the 515 nm and 633 nm lights, and larger gaps between their tip plasmon bands and the excitation wavelengths. As the contribution efficiency of hot electrons involved in energy conversions is typically within 1% [47], these subtle but existing spectral differences indicate that at resonance condition between illumination light and plasmon band of Au@Ag nanostars, one can achieve a higher conversion rate of DMAB from *p*-ATP.

**Figure 6:**
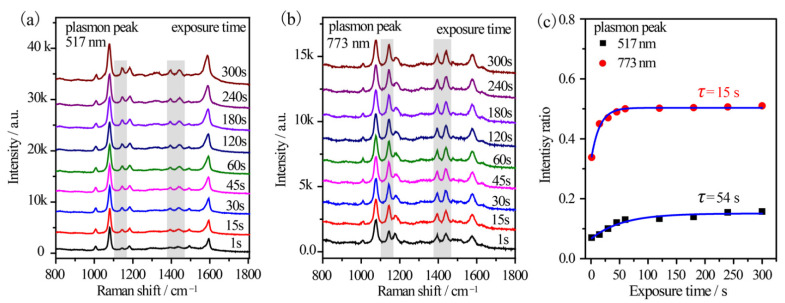
Time-dependent SERS spectral evolutions of (**a**) sample 5#′ with 517 nm tip plasmon mode and (**b**) sample 2#′ with 773 nm tip plasmon mode, which were illuminated by a 785 nm fiber laser equipped on a portable Raman spectrometer. (**c**) The time-dependent relative intensity ratios between the 1440 cm^−1^ shift of DMAB and the 1080 cm^−1^ shift of *p*-ATP.

Furthermore, the reaction kinetics were in-situ investigated by using a 785 nm excitation from a portable Raman spectrometer. An output power of 10% was used to simulate the monochromatic light. Sample 2#′ and 5#′ were investigated considering their distinct plasmon variation. For sample 5#′, the time-dependent SERS spectra of *p*-ATP were collected (Figure 6a). The vibrational fingerprints of DMAB at 1148 cm^−1^, 1390 cm^−1^, 1440 cm^−1^, and 1578 cm^−1^ shifts were very weak at exposure of 1 s and enhanced gradually with the elongation of the exposure duration. The initial *R_I_* value was ~0.07 and it gradually enhanced to 0.16 after 300 s exposures (Figure 6c). The gradually increased intensity ratio indicated the formation of few DMAB molecules on the surface of the Au@Ag core–shell nanostars as the illumination progressed. This was also confirmed by the broaden peak at 1593 cm^−1^ shift (δ(NH_2_) of *p*-ATP) due to the appearance of a shoulder at 1578 cm^−1^ (ring C–C stretching of DMAB). However, the vibrational fingerprints of DMAB were much more remarkable in the case of sample 2#′ (Figure 6b), where DMAB molecules already formed as the *R_I_* value was 0.33 within just 1 s excitation. Subsequently, the *R_I_* value increased slightly and stabilized at ~0.51 in 5 min (Figure 6c), implying that the oxidation reaction of *p*-ATP quickly reached equilibrium. The results further indicated optimal plasmon-driven catalytic efficiency upon the resonance between the tip plasmon band of Au@Ag core–shell nanostars and the excitation wavelength. In addition, the time-dependent formation of DMAB molecules was fitted based on Poisson’s equation (I = a + b·exp(−t/τ)) and shown in Figure 6c [30]. It was calculated that τ is 54 s and 15 s for sample 5#′ and 2#′, respectively, demonstrating that the plasmon effect essentially controlled the catalytic reaction rate on the surface of the Au@Ag core–shell nanostars.

### 2.5. The Mechanism of Plasmon Mediated Oxidation of p-ATP to DMAB

**Figure 7:**
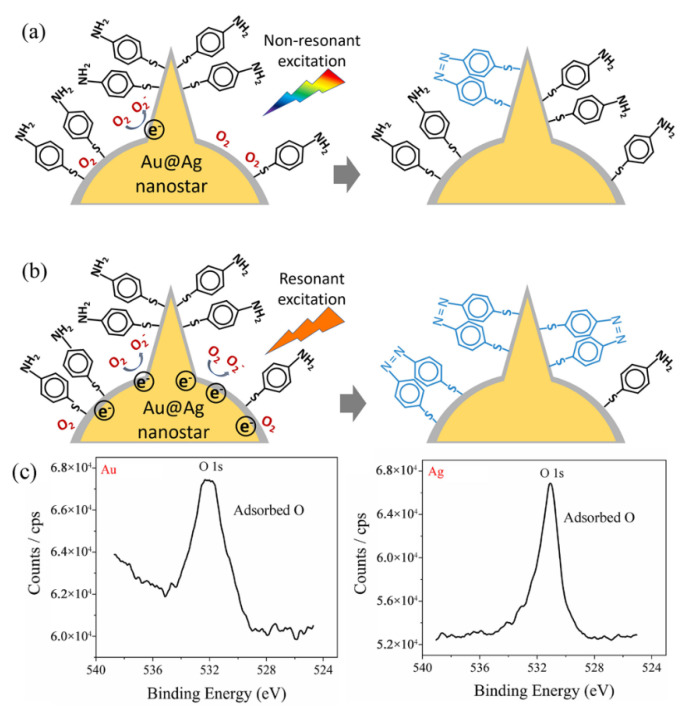
Proposed mechanism for the plasmon selective oxidation of *p*-ATP into DMAB by Au@Ag core–shell nanostars. (**a**) The case of non-resonant light excitation on Au@Ag nanostars with the tip plasmon band further from the excitation line, showing a low conversion rate to DMAB. (**b**) The case of resonant light excitation on Au@Ag nanostars with the tip plasmon mode of Au@Ag nanostars in superposition with the excitation wavelength. (**c**) XPS spectrum measured on the Au surface and Ag surface, respectively, showing adsorbed oxygen.

The mechanism of the plasmon-mediated catalytic reaction (oxidation of *p*-ATP to DMAB) on Au@Ag core–shell nanostars is schematically shown in Figure 7. For Au@Ag core–shell nanostars without resonant visible light excitation (excitation light not in superposition with the plasmon band of Au@Ag nanostars), a few hot electrons were excited by the LSPR effect. Due to the fact that Ag can adsorb more O_2_ molecules on the surface than Au (proved by XPS results in Figure 7c), the hot electrons transferred to the adsorbed O_2_ molecules, generating O_2_^−^ radicals, which then participated in the oxidation of some adsorbed *p*-ATP molecules into DMAB (Figure 7a) [30]. With resonant light illumination (excitation light in superposition with the tip plasmon band of Au@Ag nanostars), a larger number of LSPR-induced hot electrons can be excited and participate in the activation step of O_2_. Therefore, more DMAB molecules were generated on the surface of Au@Ag core–shell nanostars.

## 3. Conclusions

In conclusion, a seed-mediated synthetic approach was used to obtain Au nanostars and Au@Ag core–shell nanostars for selective plasmon-driven photocatalytic oxidation of *p*-ATP molecules. Au nanostars showed remarkable plasmonic tunability (namely, the tip mode) at Vis–NIR range (587~890 nm), while the plasmon blue shifted to 517~825 nm after being coated with ~2 nm Ag shell. The azo-coupling reaction of *p*-ATP to DMAB was employed to testify the plasmon-driven catalytic activity, while the conversion rate was evaluated by monitoring the emerged SERS fingerprints of DMAB. Results indicate that SERS spectra of *p*-ATP adsorbed on Au nanostars remained stable after 1 h of resonant excitation, suggesting poor plasmonic catalytic effect. However, the Ag shell on Au nanostars generally increased the conversion of *p*-ATP, as evidenced by the largest intensity ratio of 0.6 between the 1440 cm^−1^ shift (N=N stretching of DMAB) and 1080 cm^−1^ shift (C–S stretching of *p*-ATP). Importantly, the conversion efficiency of composite Au@Ag core–shell nanostars was dictated by the resonance between the tip plasmon mode and the excitation light. These subtle yet actual spectral evolutions of *p*-ATP show that the plasmon bands of metal nanostructures play an important role in the efficiency of plasmon-driven photocatalysis.

## 4. Materials and Methods

### 4.1. Materials

Chloroauric acid (HAuCl_4_·4H_2_O), silver nitrate (AgNO_3_), trisodium citrate, para-aminothiophenyl, sodium borohydride, N,N-Dimethylformamide (DMF), ethylene glycol, polyvinylpyrrolidone (PVP k15, Mw ≈ 10,000), ethanol, and deionized water were used for all preparations.

### 4.2. Synthesis of Au Nanostars

The Au nanostars were fabricated by Au seed-mediated approach. To synthesize Au seeds, aqueous solutions of chloroauric acid (1.2 mL, 20 mM), trisodium citrate (2 mL, 38.8 mM), and sodium borohydride (1 mL, 0.075 wt%) were injected into a beaker in turn, and stirred at 25 °C overnight (10 h). The final Au seed solution showed a light claret color, and was stocked for further use. Afterwards, aqueous solutions of chloroauric acid (0.25 mL, 50 mM), PVP k15 (1.5 g), and Au seed solution (45~750 μL) were added into DMF (15 mL) in turn, and agitated overnight to obtain transparent and blue solutions, indicating the formation of Au nanostars. Again, the size of Au nanostars can be tuned by controlling the volume of the injected Au seeds. The products were mixed with ethanol (volume ratio 1:1), separated by centrifugation (6000 rpm, 40 min), and redispersed in DMF. After three cycles, the Au nanostars dispersed in DMF were obtained.

### 4.3. Synthesis of Au@Ag Core–Shell Nanostars

The above achieved Au nanostars were sedimented by centrifugation and redispersed in ethylene glycol (1 mL). Then, the Au nanostars were mixed with ethylene glycol (5 mL) in a round-bottom flask and heated to 150 °C with a stirring speed of 300 rpm. Ethylene glycol solution of silver nitrate (0.125 mL, 16 mg/mL) was injected and let to react for 40 min. The products were separated in similar way as that for Au nanostars. The final Au@Ag core–shell nanostars were stocked in DMF or ethanol.

### 4.4. Structural Characterizations

Field emission-SEM images were taken with a JEOL JSM-7600 scanning electron microscope with an accelerating voltage of 15 kV. TEM image was taken with a JEOL JEM-2100F transmission electron microscope (Tokyo, Japan). The UV–Vis–NIR absorbance spectra were measured by Shimadzu UV-2550 spectrometer equipped with an integrating sphere (UV 2401/2, Shimadzu, Tokyo, Japan). X-ray photoelectron spectroscopy (XPS) was collected on Thermo ESCALAB 250 (Waltham, MA, USA). To investigate the plasmon-mediated catalytic effect from the Au nanostars and Au@Ag core–shell nanostars, aliquots of the nanostar suspensions (10 μL, ~1 mg/mL) were mixed with *p*-ATP solution (10 μL, 10^−5^ M) and sonicated. The mixed solution (5 μL) was dried on a silicon chip (3 × 3 mm^2^) for further light radiation and Raman measurements. An Xe light source (300 W) equipped with a monochromator was used to illuminate the samples. The power density of the emergent lights projected on the samples was tuned at 2 mW/cm^2^. SERS spectra were collected with a portable Raman spectrometer (portable B&W Tek *i*-Raman Plus, 785 nm, Newark, DE, USA).

## Data Availability

The data presented in this paper are available on request from the corresponding author.
